# The Surgical Treatment of Infective Endocarditis: A Comprehensive Review

**DOI:** 10.3390/diagnostics14050464

**Published:** 2024-02-20

**Authors:** Arian Arjomandi Rad, Alina Zubarevich, Anja Osswald, Robert Vardanyan, Dimitrios E. Magouliotis, Ali Ansaripour, Antonios Kourliouros, Michel Pompeu Sá, Tienush Rassaf, Arjang Ruhparwar, Peyman Sardari Nia, Thanos Athanasiou, Alexander Weymann

**Affiliations:** 1 Medical Sciences Division, University of Oxford, Oxford OX3 9DU, UK; 2 Department of Surgery and Cancer, Imperial College London, London SW7 5NH, UK; robert.vardanyan1@nhs.net (R.V.);; 3 Department of Cardiothoracic Surgery, Maastricht University Medical Centre, 6229 HX Maastricht, The Netherlands; 4 Department of Cardiothoracic, Transplant and Vascular Surgery, Hannover Medical School, 30625 Hannover, Germany; alina.zubarevich@gmail.com (A.Z.); anja.k.osswald@gmail.com (A.O.);; 5 Department of Cardiothoracic Surgery, University of Thessaly, 38221 Larissa, Greece; dimitrios.magouliotis.18@alumni.ucl.ac.uk; 6 Department of Cardiothoracic Surgery, John Radcliffe Hospital, Oxford OX3 9DU, UK; ali.ansaripour@ouh.nhs.uk (A.A.); antonios.kourliouros@ouh.nhs.uk (A.K.); 7 Department of Cardiothoracic Surgery, UPMC Heart and Vascular Institute, University of Pittsburgh, Pittsburgh, PA 15224, USA; 8 Department of Cardiology, West German Heart and Vascular Center Essen, University Hospital of Essen, University Duisburg-Essen, 45138 Essen, Germany; tienush.rassaf@uk-essen.de

**Keywords:** infective endocarditis, cardiac surgery, valve endocarditis

## Abstract

Infective endocarditis (IE) is a severe cardiac complication with high mortality rates, especially when surgical intervention is delayed or absent. This review addresses the expanding role of surgery in managing IE, focusing on the variation in surgical treatment rates, the impact of patient demographics, and the effectiveness of different surgical approaches. Despite varying global data, a notable increase in surgical interventions for IE is evident, with over 50% of patients undergoing surgery in tertiary centres. This review synthesizes information from focused literature searches up to July 2023, covering preoperative to postoperative considerations and surgical strategies for IE. Key preoperative concerns include accurate diagnosis, appropriate antimicrobial treatment, and the timing of surgery, which is particularly crucial for patients with heart failure or at risk of embolism. Surgical approaches vary based on valve involvement, with mitral valve repair showing promising outcomes compared to replacement. Aortic valve surgery, traditionally favouring replacement, now includes repair as a viable option. Emerging techniques such as sutureless valves and aortic homografts are explored, highlighting their potential advantages in specific IE cases. The review also delves into high-risk groups like intravenous drug users and the elderly, emphasizing the need for tailored surgical strategies. With an increasing number of patients presenting with prosthetic valve endocarditis and device-related IE, the review underscores the importance of comprehensive management strategies encompassing surgical and medical interventions. Overall, this review provides a comprehensive overview of current evidence in the surgical management of IE, highlighting the necessity of a multidisciplinary approach and ongoing research to optimize patient outcomes.

## 1. Introduction

Infective endocarditis remains one of the most significant and severe complications affecting the cardiac valvular apparatus [1]. If left untreated, the mortality rate associated with IE remains prohibitive, being fatal in almost all cases [2]. Even at experienced tertiary centres, the treatment of IE has been reported to have in-hospital mortality rates ranging from 10% to 20% and 1-year mortality rates of up to 40% [3]. The scope for surgical intervention in IE has been expanding, with both American and European guidelines supporting the role of surgery in cases of complicated left-sided infective endocarditis [4,5].

The shifting global epidemiology has included an increasing number of patients presenting with IE in the developing world, and a subsequent higher number of surgical procedures for the management of this condition. This shift has meant that most patients present at an older age and with more comorbidities, including an increased number of implanted devices, such as pacemakers and defibrillators. In recently published studies, the number of patients receiving surgery as management for IE has been reported to be increasing across the past two decades. The European Society of Cardiology EURObservational Research Programme (ESC-EORP) European Endocarditis (EURO-ENDO) study has reported that over 50% of patients with IE underwent surgical treatment in tertiary centres [6]. However, the data on the percentage of patients undergoing surgical treatment varies from 20% to 70% based on the country and availability of cardiac surgical services, along a multitude of factors [2]. A recent nationwide study in Denmark showed that only 22.5% of patients with IE underwent cardiac surgery [7]. The reflection of the heterogeneity between the reported data among countries and the lack of consistency in evidence quality has resulted in guidelines proposing recommendations based on studies which might not be truly representative of the overall population.

**Figure 1:**
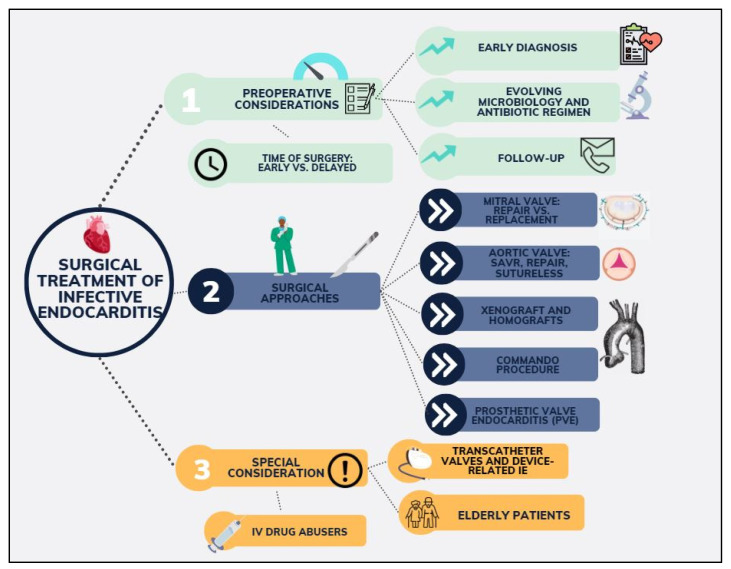
Main topics of focus on infective endocarditis covered in the manuscript.

Despite the heterogeneity in the data, it has become clear that the mortality and morbidity outcomes for patients undergoing surgery have been improving over the years, underlying the increasingly important role of surgery. Due to the high complexity of IE patients, successful management requires a multidisciplinary approach which has been recommended in all recent guidelines, with a potentially important role in early surgical intervention [4,5,8]. Nevertheless, successful operative and long-term outcomes will be multifactorial, with a special focus on the type of operation offered, the operative technicalities, and the experience of the centre. It is recommended that surgical treatment should be offered to patients with heart failure, invasion with paravalvular abscess, systemic embolization, prosthetic valve endocarditis, unresolvable sepsis, and large vegetations. In response to the evolving landscape of IE management and the ongoing debates surrounding optimal surgical strategies, this review aims to offer a comprehensive analysis of the existing literature and the latest evidence on the surgical treatment of IE (Figure 1). Our objective is to meticulously examine the entire continuum of care for IE patients—from preoperative evaluation to postoperative management—while emphasizing the surgical techniques employed, the challenges encountered in managing high-risk groups, and the gaps in current evidence that warrant further investigation. By doing so, we aspire to contribute to the refinement of surgical protocols and the enhancement of patient care in this complex and challenging field.

## 2. Materials and Methods

The authors conducted a focused review of the literature on the surgical treatment of infective endocarditis. They searched the Medline, EMBASE, and Google Scholar databases using keywords such as infective endocarditis, surgical treatment, valve replacement, valve repair, and root replacement, up to July 2023. They also examined the reference lists of relevant articles to identify any additional studies. Only articles that were deemed pertinent and dealt with the aforementioned keywords were included in the review.

## 3. Results

### 3.1. Preoperative Considerations

#### 3.1.1. Treating the Patient, Not the Disease: Aetiology, Diagnosis, Antimicrobial Treatment, and Follow-Up

Successful surgical outcomes could only be achieved if all the aspects of the disease are addressed, from diagnosis to follow-up post-surgery. Current urgent or emergent indications for the surgical treatment of endocarditis irrespective of antimicrobial duration remain IE with heart failure, uncontrollable infection, and the prevention of embolism. Cardiac surgeons should remember that treating the patient and not the disease remains essential, and cases should be discussed in a multidisciplinary team, namely the “Endocarditis Team”. Successful surgical outcomes could only be achieved if all the aspects of the disease are addressed, from diagnosis to follow-up post-surgery. In clinical practice, early diagnosis remains essential and should be carried out using the modified Duke criteria, based on clinical, microbiological, serological, and echocardiographic findings. It must be noted that the sensitivity of the Duke-modified criteria remains around 80%, with early cases of IE, especially in PVE and device IE, being more difficult to diagnose [4]. In these cases, computed tomography or cardiac magnetic resonance should be considered. Patients often present with a wide range of symptoms, with around 90% having fever, poor appetite, and shivering, and an audible murmur can be found 85% of patients. Transthoracic echocardiography (TTE) should be performed upon clinical suspicion of IE, followed by a transoesophageal echocardiogram. In cases of PVE, the diagnosis can be aided by positron emission tomography and polymerase chain reaction assays. Generally, antibiotic treatment should be guided by the culture and sensitivities of organism, with general uncomplicated cases requiring 2 weeks of penicillin or ceftriaxone with gentamicin or netilmicin [4]. Postoperatively, antibiotic prophylaxis should generally be continued for 6 weeks from the day of surgery [9]. Intraoperative samples of valve tissue should be sent to the laboratory, with positive cultures indicating a need for discussion with microbiology and the potential prolongation of antibiotics. Endocarditis prophylaxis should be considered for patients with PVE undergoing future high infective risk procedures, including dental procedures. Follow-up should generally take place at 6 weeks, and include among other investigations a TTE, with further follow-up appointments being guided by the progress of the individual patient.

#### 3.1.2. Shifting Microbiology in IE

As patient risk factors evolve, the microorganisms causing IE also change. While Streptococcus and Staphylococcus species collectively cause around 80% of cases, their prevalence varies regionally and has changed over time [10]. The rise of healthcare-associated IE has resulted in an increase in the incidence of Staphylococcus aureus and coagulase-negative staphylococci, while the proportion of IE caused by viridans group streptococci has decreased. Enterococci have become the third leading cause of IE. Gram-negative and fungal pathogens are rare causes of IE and are typically associated with healthcare-acquired infections. Blood culture-negative IE constitutes approximately 10% of cases [10]. What is referred to as true culture-negative IE is caused by microorganisms that are challenging to isolate. However, dedicated assays such as serological testing and PCR analysis of blood or tissue biopsy samples can identify a potential pathogen in around 60% of these cases [11]. The underlying causes of culture-negative IE vary based on epidemiological factors, with common pathogens including *Brucella* species, *Coxiella burnetii*, *Bartonella* species, and *Tropheryma whipplei* [11]. A recent Danish nationwide analysis of 4213 patients with IE identified *S. aureus* to be the organism linked with the highest in-hospital mortality, followed by *Enterococcus* and *Streptococcus* species [12].

#### 3.1.3. Time of Surgery

Timing of the surgical intervention to address infective endocarditis has been a matter of debate for the past few decades, with contrasting evidence being published. As the role of surgery has been expanding and has been proven to provide significant morbidity and mortality benefit to IE patients, the timing of surgery has become of crucial importance. Generally, early surgery is indicated for IE in heart failure (HF) [4,5]. Indeed, congestive cardiac failure has been recognized to have the greatest negative prognostic effect in patients with IE. Early cardiac valve surgery in patients with IE has been associated with significantly lower mortality and morbidity rates. Revilla et al. [13] reported surgery for IE and congestive cardiac failure to be performed in 72% of patients, currently representing the most prevalent indication for surgery in IE patients. Nadji et al. [14] recently reported on a series of 259 patients, of whom 46% received early surgical intervention. The researchers reported a perioperative mortality of 10%, with early surgery being an independent predictor for reduced mortality at 1-year follow-up [HR 0.45 (0.22–0.93); *p* = 0.03] [14]. Early studies for the 1970s and 1980s reported a mortality reduction of 50 to 80% in patients with HF receiving surgery for IE [15].

Whilst the benefit of early surgical intervention has been well established in the context of IE and HF, the debate remains open with regards to neurological events and cerebrovascular accidents in patients suffering from IE. Due to vegetative embolism, cerebrovascular accidents have been reported in 20–40% of patients with IE [16]. Kang et al. [17] in the Early Surgery versus Conventional Treatment in Infective Endocarditis (EASE) trial compared clinical outcomes in patients suffering from left-sided IE and at high risk of embolism who underwent either early surgery (37 patients) or conventional surgery (30 patients). Although, at six months no difference between early (within 48 h of randomization) and conventional surgery (during initial hospitalization) was found in terms of mortality, a significantly lower incidence of composite outcomes of death, embolic events, and reoccurrence of IE was found (3% in the early group vs. 28% in the conventional group; *p* = 0.02). The study elucidated the benefit of early surgery in patients with IE and large vegetation at risk of systemic emboli.

It must be noted that the EASE study excluded patients suffering from a major stroke. In general, although robust evidence is lacking, cardiac surgical intervention in IE should not be delayed when a CT head excludes a haemorrhagic stroke and is generally not contraindicated in patients after an ischaemic stroke (assuming the neurological deficit is not severe). In the setting of a stroke, a bioprosthetic or non-mechanical valve is recommended to avoid the need for lifelong anticoagulation, thus decreasing the risk of haemorrhagic transformation.

### 3.2. Surgical Approaches to Infective Endocarditis

#### 3.2.1. Isolated Valve: Valve Repair or Replacement?

##### Mitral Valve

The feasibility and improved long-term outcomes of mitral valve repair in degenerative mitral valve regurgitation resulting from chordal rupture or leaflet prolapse have been recognized in multiple large-scale studies, with current multi-regional guidelines recommending the procedure whenever feasible [18]. In the context of infective endocarditis, the debate over repair versus replacement has been ongoing, with the main concerns regarding durability and long-term outcomes. MV repair in the setting of IE involves a spectrum of techniques, chosen based on the lesion’s location, the extent of leaflet destruction, and the presence of associated complications such as paravalvular abscess. The resection technique, a cornerstone of MV repair, demands the removal of sufficient leaflet tissue to ensure the complete eradication of the infectious process. However, the specific approach varies significantly—lesions on the anterior leaflet, commissural lesions, and scenarios involving extensive leaflet destruction or paravalvular abscess may necessitate distinct and often more complex repair techniques. For instance, annuloplasty, which involves the use of a prosthetic ring to provide leaflet coaptation, limit suture tension, and prevent further annular dilation, is pivotal for achieving stable long-term surgical outcomes. Considerations on the choice of the annuloplasty ring should be made, keeping in mind complications such as ring dehiscence, reported to be higher among rigid rings as opposed to flexible rings [19]. Additionally, the utilization of the autologous pericardium as an alternative material for annuloplasty in patients with active IE represents an adaptation to the unique challenges posed by the infectious milieu.

The feasibility of performing mitral valve repair for IE was first put forward by Dreyfus et al. in 1990 in a series of 40 patients, reporting in-hospital mortality rates of 2.3% for operated patients [20]. In 2007, Ferringa and colleagues conducted a systematic review and pooled analysis of 24 studies, illustrating that MV repair in IE was associated with significantly lower rates of in-hospital and long-term mortality, as well as lower re-operation rates, cerebrovascular accidents, and recurrent endocarditis when compared to MV replacement [21]. Despite these results, concerns about the retrospective and small size of these studies remained, with most of the patients undergoing replacement being older and more comorbid. A nationwide cohort study conducted in Taiwan in 2018 evaluated 1999 patients with IE who underwent either MV repair or replacement during a 13-year period [22]. The investigators found that over a median follow-up period of 4.8 years, patients receiving MV repair had significantly lower in-hospital mortality, follow-up mortality, and perioperative complications. Interestingly, the study also highlighted that the benefits of MV repair were more apparent than when carried out in high-volume centres. The impact and effect of the centre experience in mitral valve repair on outcomes have been well documented in the general cardiac surgical literature [23], illustrating the importance of the learning curve and the lack of standardized results among centres with different levels of expertise in the procedure [24]. The beneficial effects of MV repair have been attributed to the maintenance of the mitral valve apparatus and left ventricular function [25]. Furthermore, MV repair avoids or minimizes the introduction of prosthetic material which is brought in by MV replacement. Indeed, MV replacement with a mechanical valve increases the risk of thromboembolic events, as well as introducing patients to anticoagulants, while bioprosthetic valves are prone to inevitable structural deterioration [26]. It must be noted that the feasibility of repair must be taken into the context of the extent of valvular and extra-valvular destruction, with careful intraoperative assessment of the valve following careful debridement [27]. Extensive involvement and damage of the anterior mitral leaflet and perivalvular structures and annular abscess formation might be considered as complicating the feasibility of repair [27,28], especially for less experienced centres.

The lack of standardization in MV repair techniques must be highlighted and can largely be attributed to the variability in clinical presentations and the extent of complications associated with valve IE. This diversity necessitates a high degree of customization in surgical strategies, underscoring the importance of surgical expertise and experience in determining the most appropriate course of action. Furthermore, it is imperative to acknowledge the scarcity of comprehensive long-term data on patients who have undergone MV repair for IE. Information regarding long-term valve function, the recurrence of endocarditis, and overall patient outcomes remains limited, highlighting a significant gap in our current understanding. This lack of data underscores the urgent need for systematic follow-up studies and registries to better inform clinical practice and improve patient care strategies in this challenging domain.

##### Aortic Valve

**Figure 2:**
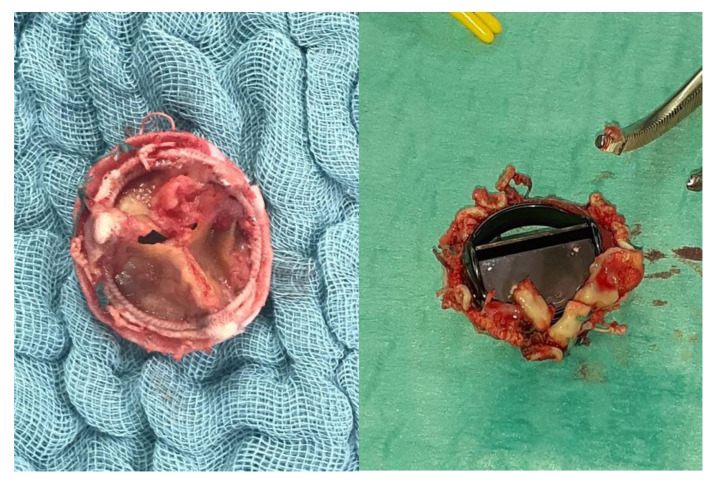
Bioprosthetic (**left**) and mechanical (**right**) PVE.

Aortic valve (AV) surgery in the setting of infective endocarditis has been traditionally almost exclusively comprised of valve replacement, therefore exposing patients to the established risk of prosthesis-associated complications of IE reoccurrence (Figure 2). IE has been considered as one of the risk factors leading to aortic regurgitation [28], and its treatment up to today has been recommended to follow the traditional decision-making as per non-IE aortic disease. However, over the past decade, AV repair has emerged as a possible option for patients with IE [29]. Mayer et al. in 2012 published a small series of 100 patients receiving either AV repair or replacement for AV IE, with repair receiving autologous or pericardial patches for cusp and root lesions [29]. The group found survival and freedom from moderate–severe aortic regurgitation at 5 years to be significantly higher in the AV repair group when compared to the replacement group, with a similar rate of reoperation. Most recently, Solari and colleagues reported on the outcomes of AV repair in 47 patients operated on from 1998 to 2017 for IE [30]. The group found AV repair to be a feasible approach, with a 5- and 10-year mortality of 89 ± 9.4% and 76.6 ± 16%, respectively. In general, the group also found patients with a bicuspid aortic valve to have significantly higher rates of reoperation when compared to tricuspid aortic valve patients. Several limitations exist and considerations must be made when repair is considered as an approach to the AV in infective endocarditis. Firstly, the reports on repair remain extremely limited to small-centre retrospective studies which do not provide data representative of the overall population and are intrinsically prone to selection bias. Secondly, it must be noted that aortic valve repair remains a feasible option only for IE limited to the aortic valve leaflets, with any extra-valvular extension of the infection, including aortic annulus involvement, making repair an unfeasible option. While exploring the potential of AV repair in IE offers intriguing insights, it is crucial to acknowledge that such surgical techniques are not widely adopted across most surgical centres and may not be within the scope of standard practice for the majority of reasonably trained and experienced adult cardiac surgeons. The advocacy for these less conventional approaches should be viewed with caution, given their limited study and the variability in reported outcomes. As such, these methods should be considered as provocative perspectives that may stimulate further research rather than established practices. Ensuring patient safety and adhering to evidence-based standards remain paramount in the surgical management of IE.

#### 3.2.2. Sutureless Valves: An Option Yet to Explore

Sutureless aortic valves have emerged over the last decade as a feasible option to the treatment of aortic valve disease, allowing a broader and more standardized implementation for minimally invasive surgery, as well as leading to a reduction in cross-clamp (CC) and cardiopulmonary bypass (CBP) times [31,32]. The literature with regards to the implementation of sutureless AV in infective endocarditis remains extremely scarce, mainly limited to small cases series and retrospective single-centre studies. Although the deployment of these valves in IE is still considered “off-label”, they could potentially be an option, especially when reduced CC and CBP times are desirable for high-risk patients. Zubarevich et al. reported on 13 high-risk patients receiving sutureless AV replacement with Perceval S in the context of IE, with 8 of the patients needing a concomitant cardiac procedure and 8 also being redo-operations [33]. They illustrated CBP and CC times of 89.8 ± 33.6 and 59.1 ± 27.8 min, respectively. The group also showed mean postoperative gradients of 8.1 ± 4.8 mmHg. Their data illustrated that sutureless valves could be considered as a feasible option in high-risk patients, who would benefit from a decrease in operating time. Their implantation assumes an intact annulus which is not always the case if one needs to address root abscesses with patch reconstruction. Further research into the safety and clinical feasibility of these valves in the context of IE is awaited.

#### 3.2.3. Aortic Homograft in IE

The use of aortic homografts as an alternative to the traditional aortic valve replacement in the context of IE has been proposed to be an optimal option by multiple groups, especially in cases of extensive periannular abscess and redo-operations [34]. The theoretical advantage of homografts as opposed to conventional prosthetic aortic valves remains in the lack of synthetic material and the use of biological tissue. One of the main benefits of an aortic homograft is the additional tissue provided with the homograft around its annulus that can be used to reconstruct defects in the root and LVOT as well as in the aortomitral curtain (making use of the homograft’s anterior mitral leaflet when it comes with one). Furthermore, the role of aggressive debridement and root replacement as opposed to simple AV replacement has been proposed to provide superior outcomes. In general, when over one-third of the annulus is involved, then root replacement is considered a better option than valve replacement [35]. Aortic homografts have also been shown to provide extensive antibacterial properties [36]. Nevertheless, due to a lack of randomized controlled trials, their technical-operative intricacies, and the lower availability, the use of homografts for AV infective endocarditis has been decreasing. Kim et al. in 2016 reported that a significantly higher proportion of patients with IE were being treated with a homograft in cases of abscess formation and infection with methicillin-resistant Staphylococcus, when compared to mechanical valves or xenografts [37]. Numerous studies have been continuously reporting on the long-term outcomes of homografts. Arabkhani et al. [38] on a 27-year follow-up illustrated a low incidence of re-infection (2.2%), which was also supported by results from Fukushima et al. [39] showing rates of 5.5% at 5 years. Freedom from reoperation was also reported to be excellent with homografts with results from Musci et al. [34] showing a 10-year rate of 92.9% in patients with a native aortic valve IE. Similar results were also reported by Solari et al. [40] with a freedom from reoperation at 10 years being 86.3 ± 5.5%. Long term survival with aortic homografts has also been shown to be excellent. Yankah et al. [41] illustrated 10-year survival to be 91%, while Perrotta et al. [42] showed a 5-year survival of 88%. Musci et al. [34] reported the 5-year survival following native aortic replacement with a homograft to be 66.5%. Even in high-risk patients with reoperative aortic root and proximal aortic arch involvement, Preventza et al. [43] showed a follow-up survival rate at a median of 2.5 years to be 65.7% and a freedom from reinfection rate of 100%. Despite the positive outlook and results presented by multiple studies, some recent reports have presented contrasting evidence. Jassar et al. [44] reported a 10-year follow up of 134 patients, showing no difference in rates of reinfection, readmission, in-hospital mortality, and major complications between homograft, mechanical, or bioprosthetic aortic valve replacement in IE. Of note, the study did not elucidate whether an aortic annular abscess was present or not in their patients. Overall, aortic homografts have been reported to start exponentially deteriorating after 10 years following their placement [45,46]. While the reported outcomes of aortic homografts in IE, including the spectacular freedom from reoperation rates, underscore their potential benefits, it is imperative to contextualize these findings within the broader experience of structural failures noted in wider clinical practice. The discrepancy between reported long-term success and the actual higher structural failure rates observed with homografts may reflect a reluctance to offer reoperations to patients experiencing a progression of aortic insufficiency, the primary pathology associated with homografts and stentless xenografts, rather than aortic stenosis. This hesitancy is often due to the high-risk nature of reoperative aortic root surgery, particularly in patients who have previously received a homograft or xenograft. Such considerations highlight the need for a cautious interpretation of the data and a balanced discussion on the viability of homografts as a treatment option in IE.

#### 3.2.4. Stentless Xenografts for IE

One of the main contemporary issues relating to xenografts, whether stentless or stented, has been the rate of surgical valve deterioration, which, similarly to homografts, has been reported to start around 8–10 years post-implantation. The treatment of aortic root endocarditis remains complicated, especially when a periannular abscess is present. The search for the best valve has been ongoing for the last couple of decades, with varying degrees of evidence available to support each treatment approach. Stentless aortic root replacement enables difficult reconstructive surgery to be carried out at the outflow tract and the annular level. Recently, a prospective randomized controlled trial from the Royal Brompton and Harefield Hospitals group compared the long-term outcomes of homografts versus Medtronic Freestyle in 166 patients [47]. Overall survival at up to 20 years of follow-up showed no significant difference between the two groups, with similar findings in terms of freedom from aortic valve reoperation (67.9 ± 8.8% with Freestyle vs. 67.2 ± 10.3% with homograft; *p* = 0.74) [47]. Clemence et al. [48] obtained data from the Society for Thoracic Surgeons database for 265 patients with aortic valve IE who underwent AVR between 1998 and 2017. At 10 years postoperation, the stentless graft group reported 98% freedom from endocarditis, versus 88% in the stented group, while the risk of reoperation was 12.4% in the stented group versus only 3.4% in the stentless valve group. Easo et al. [49] propensity-matched stentless Medtronic Freestyle with other tissue valves for full root endocarditis. The group reported a freedom from reoperation with the Medtronic Freestyle to be 97.2% at a median of 2.7 years postoperatively. In a separate study by Szczechowicz et al. [50], the same group compared long-term outcomes of 80 patients undergoing either root replacement with Medtronic Freestyle, homograft, bioprosthetic, or mechanical valves. Homograft and stentless valves demonstrated the best mean survival rates (13.7 years and 8.1 years) followed by the biological and mechanical stented valves (2.8 and 1.4 years respectively). These data remain controversial as the evidence with regards to stentless xenografts remains scarce. Schaefer et al. [51] compared the stented Carpentier-Edwards Perimount valve with the Sorin Freedom Solo Stentless aortic valve in 154 patients, demonstrating at 6 years a significantly lower SVD and higher survival rate with the stented valve when compared to the stentless valves. Nevertheless, haemodynamic superiority was achieved with the stentless valve. The evidence in the field of stentless xenografts remains scarce due to their lower uptake; however, data from expert centres appear promising. The advent of specially treated bioprostheses such as the Inspiris Resilia that claim increased freedom from structural deterioration to their conventionally treated counterparts may have a place in the treatment of IE in the younger population. While there is supportive clinical data in the general group up to 7 years—Commence trial [52]—the evidence in IE is still sparse. Furthermore, the surgical complexity inherent in performing a stentless root replacement, be it a xenograft or homograft, compared to a simpler aortic valve replacement, is difficult to justify in light of the increased morbidity and complication rates. This reconsideration is underscored by the realization that the alleged long-term benefits, such as more durable hemodynamic performance and avoidance of lifelong warfarin therapy, often do not materialize in real-world clinical settings. Accordingly, the use of a xenograft, particularly in the context of aortic root endocarditis complicated by extensive root abscess, may be considered only under specific circumstances, such as the unavailability of homografts, underscoring a need for a cautious and evidence-based approach to selecting surgical interventions in IE.

#### 3.2.5. Extensive Aortic Root or Mitral Valve Infective Endocarditis: Commando

**Figure 3:**
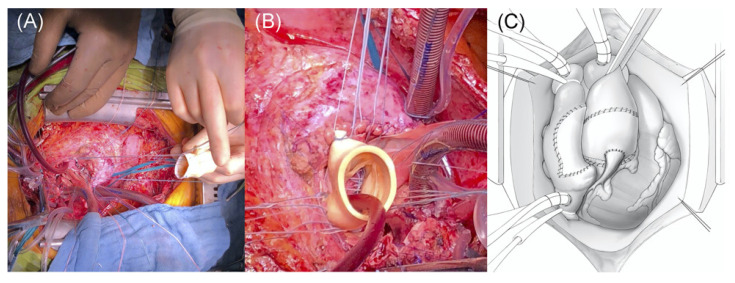
Commando Procedure–Essen Modification. (**A**) Conventional implantation technique of a stentless aortic root prosthesis; (**B**) lowering of the stentless aortic root prosthesis into the native aortic root. (**C**) Final postoperative result: implanted aortic root prosthesis and right atrium closed with the anterior aspect of the pericardium patch. Reproduced from Zubarevich et al. [57] with permission from the authors.

In severe cases of infective endocarditis, involvement of the intervalvular fibrous body (IVFB) poses a significant clinical challenge for cardiac surgeons. The IVFB is constituted by the interleaflet trigone between the left and non-coronary aortic sinuses, the roof of the left atrium and the anterior mitral leaflet. Surgical reconstruction of the IVFB could be a serious surgical task, especially in the context of extensive IE. The mortality associated with this procedure has remained high since its introduction in 1979 by David and colleagues [53], with a 1-year rate ranging between 20% and 30% and a significant rate of intraprocedural complications [54]. As a result of the difficulties encountered by several groups performing IVFB reconstruction in conjunction with mitral and aortic valve replacement, this procedure, also named “Commando” or “David technique”, has received multiple modifications across the years. Furthermore, due to the low incidence of this clinical scenario, only a few small-scale studies have been published. Forteza et al. [55] presented their data from 1997 to 2013 on 40 consecutive patients undergoing IVFB reconstruction, 26 of whom were due to IE. The data for patients with IE at 5 and 10 years reported a freedom from reoperation of 84.6% and 76.9%, respectively, and a survival rate of 57.7% and 50%, respectively. The authors concluded that due to its high risk, the procedure remains a safe and feasible alternative for patients where no alternative option is available, underlining the need for continuous surveillance due to the risk of late dehiscence. Indeed, David et al. [53] had also noted an increased rate of re-exploration for bleeding in their early studies, most likely resulting from a contribution from multiple factors, including fragile tissues, difficulty in accessing posterior suture lines, and the urgent nature of the surgical procedure. Davierwala et al. [54] reported on 25 patients undergoing the Commando procedure, resulting in a 30-day mortality rate of 32%, with the major cause of mortality being major bleeding due to poor patch anchoring. Most recently, David et al. in 2022 [56] reported the long-term outcomes of the Commando procedure, in 182 consecutive patients operated on from 1985 to 2020, and the operative mortality was 13.2% and 10- and 20-year survival were 51.1% and 23.7%, respectively. The rate of the reoperation for this procedure, however, remains high, at around 50% in the first 5 years across the mentioned studies. Recently, the group at Essen proposed a modified procedure renamed the Essen–Commando [57] (Figure 3), involving the use of a Medtronic Freestyle stentless aortic root prosthesis for replacement. The group proposes that due to the flexibility, this prosthesis is able to offer optimum haemostatic activity and haemodynamic performance.

#### 3.2.6. Prosthetic Valve Endocarditis

Prosthetic valve endocarditis (PVE) constitutes one of the most severe forms of IE, with an increased hospital mortality rate of 20–40% [4]. The incidence of PVE has been increasing over the years with an increased number of surgically implanted prosthetic valves, affecting mechanical and bioprosthetic valves equally. The current evidence around PVE reports that it accounts for around 10 to 30% of cases of IE [58], often correlated with difficulty in diagnosis and a lower prognosis when compared to other forms of IE.

Surgical management of PVE includes extensive debridement and the removal of any prosthetic material and decalcification. Debate has been ongoing regarding the best prosthesis to deploy in cases of PVE, with homografts and stentless xenografts being generally preferred to standard stented aortic valves. David et al. [59] published a series of 383 patients undergoing surgery for IE and found PVE to be an independent predictive factor for increased mortality and worsened outcomes in patients undergoing surgical intervention. Wang et al. [60] published a multicentre propensity-matched study of an international cohort of 367 patients with PVE. They illustrated that 42% of the patients underwent surgical intervention, with their results trending toward an additional benefit provided by surgery. Their results demonstrated that brain embolization and *Staphylococcus aureus* were associated with in-hospital mortality. Similar results were also reported by Manne et al. [61], who in a series of 428 patients undergoing IE surgery, of whom 42% had PVE, found *S. aureus* to be associated with significantly higher mortality. Nevertheless, Sohail and colleagues [62] carried out a retrospective comparison of PVE patients with *S. aureus* infection treated either surgically or medically. Their results demonstrated increased mortality with medical-treated group compared to the surgical intervention group (48% in the medical group vs. 28% in the medical group). Their results also illustrated bioprosthetic valves to be independent predictors of mortality. Recurrence and relapse of PVE occur in 5% [63] of patients that are medically treated, with a higher tendency of relapses in E faecalis infection [64] and a high mortality of 24% [63]. Surgery is indicated in relapses to eradicate the infective focus.

Overall, it could be gained that although mortality with PVE is higher than in native valves, a mortality advantage is provided by surgery when compared to medical therapy alone.

##### Transcatheter Valves, New Friends to an Old Problem for Cardiac Surgeons

**Figure 4:**
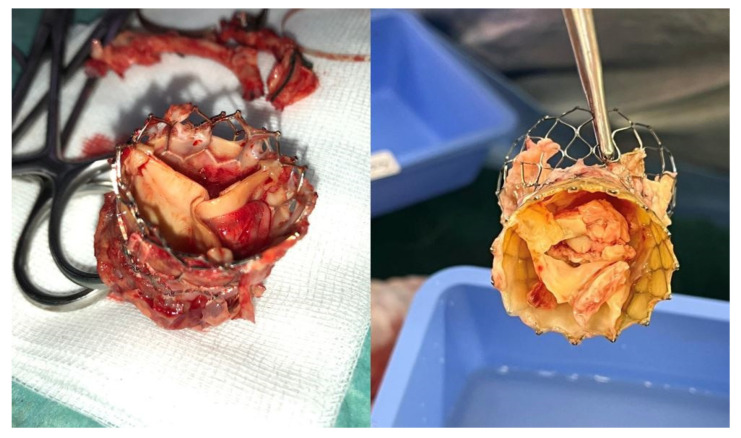
TAVI endocarditis after being explanted.

The advent and rapid expansion of transcatheter aortic valve implantation (TAVI) has introduced a new high-risk population at risk of developing PVE (Figure 4). Although TAVI has been expanding to intermediate and currently also to low-risk patients, the majority of patients treated with TAVI remain elderly and comorbid, and they were originally deemed too high risk for SAVR. The incidence of post-TAVI IE was recently investigated among the SwissTAVI registry by Stortecky et al. [65], who demonstrated the rate peri-procedurally to be 2.59, delayed-early to be 0.71, and late IE to be 0.40 events per 100 person-years. The group found *Enterococcus* species to be the most common pathogen, with patients being at increased risk of stroke and mortality. Khan et al. [66] in a recent systematic review on the topic found that among 11 studies, the mean incidence of TAVI-associated PVE was 3.25%, ranging in studies from 0% to 14%. In line with previous findings, it was found that the most common species isolated were *Enterococci*, with a high cumulative incidence of heart failure, stroke, and major bleeding at 37.1%, 5.3%, and 11.3%, respectively. The review also found that mean in-hospital mortality and follow-up mortality were 29.5% and 29.9%, respectively [66]. These data outline the increased number of patients who in the future will present with PVE, with a high risk of mortality and morbidity, with cardiac surgeons having to make difficult decisions regarding surgical intervention. Current guidelines advocate for early surgery for cases of complicated IE, especially in patients with congestive HF, paravalvular extension of the infection, and increased risk of embolism [4,5]. Nevertheless, the data about the potential benefit of surgery in TAVI PVE are lacking, being represented mostly by small retrospective series, most of which have not shown a mortality advantage with surgical intervention when compared to medical therapy alone. In cases where a surgical approach is carried out, an extensive surgical intervention should be undertaken. Zhigalov et al. [67] have proposed that improved outcomes in this high-risk population could potentially be achieved by lowering CBP and CC times through the deployment of sutureless valves, although further evidence is needed.

##### Cardiac Implantable Electronic Devices Infective Endocarditis

The incidence of Cardiac Implantable Electronic Devices (CIED) IE has been increasing with the steady growth in the deployment of pacemakers and implantable defibrillators. The management of this complication almost always entails the removal of the device, and in cases of extensive infection, the debridement of any infected lesions [2]. Complete removal of the device in the setting of IE remains necessary due to the high relapse rates related to devices left in situ. Usually, for limited-extent IE with a device, percutaneous removal of the leads could be attempted; nevertheless, the “endocarditis team” should be mindful of the potential complications, some of which being more severe like right ventricular perforation leading to cardiac tamponade. As outlined in the latest European Heart Rhythm Society (EHRA) guidelines [68], for limited-extent IE involving a device, percutaneous transvenous extraction techniques are preferred due to their lower rates of major complications and mortality at 1 and 12 months compared to open surgical approaches. These procedures are favoured even in the presence of lead vegetations larger than 10 mm, with small case series reporting satisfactory short-term outcomes despite a high incidence of pulmonary embolisms. However, the long-term outcomes of patients with large lead vegetations undergoing transvenous lead extraction remain to be fully elucidated. In cases where vegetations exceed approximately 20 mm, or when the vegetation’s friability poses a significant risk, open surgical extraction may be considered as a viable alternative. High-risk extractions, e.g., PPMs > 15 years or dual coil ICDs, should take place in an environment with the physical presence of the surgical team and perfusionists [69]. The placement of epicardial leads if surgery is indicated for the extraction of infected ones could be an option, especially for patients at higher risk of re-infections.

### 3.3. Considerations in High-Risk Groups

#### 3.3.1. IV Drug Abusers

Despite multiple worldwide efforts to decrease the rate of global intravenous drug abuse (IVDU), the rate has been rapidly rising, increasing the risk of IE and presenting cardiac surgeons with new challenges. Although most of the patients presenting with developing IE who use intravenous drugs are relatively young and less comorbid than the general cardiac surgical population, the mortality associated with IE in this group remains as high as 40%, with 60 to 70% requiring surgical intervention [70]. The patient’s clinical picture is further complicated by their non-compliance, repeated abuse of IV drugs even after IE events, and the presence of highly aggressive and resistant bacterial species generally less responsive to antibiotic treatment regimens.

Between 2007 and 2020, Zubarevich et al. [71] evaluated 24 consecutive IVDU patients who underwent valve surgery for IE, with a mean follow-up of 4.2 years, and reported a 30-day survival of 88% and 72% at three years from surgery. The rate of readmission due to recurrent IE was 20.8%, while only 58% remained free from a composite endpoint of freedom from mortality, recurrent endocarditis, re-do surgery, and postoperative stroke. Most recently, Bearpark et al. [72] compared outcomes between 55 IVDU and 455 non-IVDU patients with IE receiving surgery. Survival at 8 years post-surgery was 35% in IVDU versus 68% in non-IVDU groups. Sultan et al. [73] evaluated the use of homografts in IVDU undergoing aortic root replacement, evaluating mid-term outcomes in 138 patients and reporting an estimated 5-year survival of 43%.

The tricuspid is the most commonly affected valve in IVDUs. Complete destruction of the valve is not uncommon, and the timing and type of surgery pose significant challenges. Tricuspid valvectomy can provide an acceptable bridge to tricuspid valve replacement allowing for sterilization of the field in extensive endocarditis [74].

Generally, the preferred surgical approach to IVDU should include early and aggressive surgical intervention to limit the risk of deterioration, paired with appropriate antibiotic therapy as well as anti-addiction support and treatment.

#### 3.3.2. Surgery in the Elderly

The increased ageing world population had similarly contributed to an increase in the incidence of IE, with the elderly representing a growing portion of patients, whom cardiac surgeons will have to clinically assess and manage. Age and frailty have already been established to be a prognostic factor for mortality in IE [75,76]. The definition of “elderly” has been heterogenous across different studies, with some reporting >70 years of age as elderly while others report >80 years of age as elderly, leading to a potential heterogeneity in results. Recently a nationwide study assessed the current state of IE in Japan, one of the worlds’ fastest ageing countries. Kiriyama et al. [77] analysed a national database consisting of over 20,000 patients admitted with IE, reporting a significant increase from 19.1% to 29.7% of very elderly patients (>80 years old) during an 8-year period. The results of the analysis demonstrated that surgical intervention was associated with reduced in-hospital mortality even in the very elderly population. The rates of surgical intervention in this population remained significantly lower than the one in younger patients, being less than 10%. A separate recent nationwide analysis carried out in Sweden by Ragnarsson et al. [78] similarly found that rates of surgical intervention were decreasing with increasing age despite mortality rates being significantly lower in patients undergoing surgery when compared to medical therapy alone. In their matched population, surgery provided improved long-term outcomes (HR, 0.36; 95% CI, 0.24–0.54; *p* < 0.001) and this was found irrespective of age, although absolute mortality was higher in the very elderly. The results from the literature clearly underline an underuse of surgery in the elderly population who could potentially benefit from it. The general reluctance of offering surgical treatment in all elderly populations will need to be addressed at regional and nationwide levels through careful patient selection and a dedicated “endocarditis” team as already widely stipulated by guidelines.

## 4. Conclusions

Surgery for the treatment of infective endocarditis has been developing for the past decades, leading to improved mortality and morbidity outcomes for a severely ill patient population whose prognosis was generally considered to be poor. The varying degrees of IE cardiac structural involvement have led to the consideration of specific surgical approaches for different problems. Mitral valve repair has appeared throughout the last decade to be the preferred approach for IE affecting the mitral apparatus, when feasible. The management of aortic valve and root disease remains debatable, with varying degrees of contrasting evidence regarding the deployment of homografts and stentless and stented xenografts. Overall, it appears that stented xenografts have demonstrated a good long-term prognosis and are preferred. Nevertheless, an important role for both homografts and stentless xenografts exists in extensive IE, with long-term outcomes being shown to be optimal at least in experienced centres. Early surgery for IE is making headway especially for patients who experience concomitant heart failure. The timing of surgery in patients with neurological deficits remains a matter yet to be fully clarified. Future cardiac surgeons will be faced with an increasing burden of IE related to TAVI and implantable devices, and appropriate surgical management will have to be a key part of their armamentarium. Clinical research within the field of the surgical treatment of IE remains difficult mainly due to the emergent nature of the condition and the potential ethical questions raised in running randomized controlled trials in this setting.

## Data Availability

All data used in the manuscript are available publicly.
